# Study protocol of the B-CAST study: a multicenter, prospective cohort study investigating the tumor biomarkers in adjuvant chemotherapy for stage III colon cancer

**DOI:** 10.1186/1471-2407-13-149

**Published:** 2013-03-25

**Authors:** Megumi Ishiguro, Kenjiro Kotake, Genichi Nishimura, Naohiro Tomita, Wataru Ichikawa, Keiichi Takahashi, Toshiaki Watanabe, Tomohisa Furuhata, Ken Kondo, Masaki Mori, Yoshihiro Kakeji, Akiyoshi Kanazawa, Michiya Kobayashi, Masazumi Okajima, Ichinosuke Hyodo, Keiko Miyakoda, Kenichi Sugihara

**Affiliations:** 1 Department of Surgical Oncology, Tokyo Medical and Dental University, Graduate School, 1-5-45 Yushima, Bunkyo-ku, Tokyo, 113-8519, Japan; 2 Department of Surgery, Tochigi Cancer Center, 4-9-13 Yonan, Utsunomiya, Tochigi, 320-0834, Japan; 3 Department of Surgery, Japanese Red Cross Kanazawa Hospital, 2-251 Minma, Kanazawa, Ishikawa, 921-8162, Japan; 4 Department of Surgery, Hyogo College of Medicine, 1-1 Mukogawa-cho, Nishinomiya, Hyogo, 663-8501, Japan; 5 Department of Clinical Oncology, National Defense Medical College Hospital, 3–2 Namiki, Tokorozawa, Saitama, 359-8513, Japan; 6 Department of Surgery, Cancer and Infectious Diseases Center Komagome Hospital, 3-18-22, Honkomagome, Bunkyo-ku, Tokyo, 113-8677, Japan; 7 Department of Surgical Oncology, The University of Tokyo, 7-3-1 Hongo, Bunkyo-ku, Tokyo, 113-8655, Japan; 8 First Department of Surgery, Sapporo Medical University, South 1, West 16, Chuo-ku, Sapporo, Hokkaido, 060-8543, Japan; 9 Department of Surgery, Nagoya Medical Center, 4-1-1 San-no-maru, Naka-ku, Nagoya, Aichi, 460-0001, Japan; 10 Department of Gastroenterological Surgery, Osaka University, Graduate School of Medicine, 2-15 Yamadaoka, Suita, Osaka, 565-0871, Japan; 11 Department of Surgery, Division of Gastrointestinal Surgery, Graduate School of Medicine, Kobe University, 7-5-2 Kusunoki-cho, Chuo-ku, Kobe, Hyogo, 650-0017, Japan; 12 Department of Surgery, Osaka Red Cross Hospital, 5-30 Hudegasaki-cho, Tennoji-ku, Osaka, 543-8555, Japan; 13 Department of Human Health and Medical Sciences, Kochi Medical School Kohasu, Okou-cho, Nangoku, Kochi, 783-8505, Japan; 14 Department of Surgery, Hiroshima City Hospital, 7-33 Motomachi, Naka-ku, Hiroshima, 730-8518, Japan; 15 Department of Gastroenterology, University of Tsukuba, Graduate School of Comprehensive Human Sciences, 1-1-1 Tennodai, Tsukuba, Ibaraki, 305-8575, Japan; 16 Department of Analyses, Translational Research Informatics Center, 1-5-4 Minatojima-minamimachi, Chuo-ku, Kobe, Hyogo, 650-0047, Japan

**Keywords:** Colon cancer, Adjuvant chemotherapy, 5-FU, Personalized therapy, Cohort study, Translational research, Thymidine phosphorylase (TP), Thymidylate synthase (TS), Dihydropyrimidine dehydrogenase (DPD), Orotate phosphoribosyl transferase (OPRT)

## Abstract

**Background:**

Adjuvant chemotherapy for stage III colon cancer is internationally accepted as standard treatment with established efficacy. Several oral fluorouracil (5-FU) derivatives with different properties are available in Japan, but which drug is the most appropriate for each patient has not been established. Although efficacy prediction of 5-FU derivatives using expression of 5-FU activation/metabolism enzymes in tumors has been studied, it has not been clinically applied.

**Methods/design:**

The B-CAST study is a multicenter, prospective cohort study aimed to identify the patients who benefit from adjuvant chemotherapy with each 5-FU regimen, through evaluating the relationship between tumor biomarker expression and treatment outcome. The frozen tumor specimens of patients with stage III colon cancer who receives postoperative adjuvant chemotherapy are examined. Protein expression of thymidine phosphorylase (TP), dihydropyrimidine dehydrogenase (DPD), epidermal growth factor receptor (EGFR), and vascular endothelial growth factor (VEGF) are evaluated using enzyme-linked immunosorbent assay (ELISA). mRNA expression of TP, DPD, thymidylate synthase (TS) and orotate phosphoribosyl transferase (OPRT) are evaluated using reverse transcription polymerase chain reaction (RT-PCR). The patients’ clinical data reviewed are as follow: demographic and pathological characteristics, regimen, drug doses and treatment duration of adjuvant therapy, types and severity of adverse events, disease free survival, relapse free survival and overall survival. Then, relationships among the protein/mRNA expression, clinicopathological characteristics and the treatment outcomes are analyzed for each 5-FU derivative.

**Discussion:**

A total of 2,128 patients from the 217 institutions were enrolled between April 2009 and March 2012. The B-CAST study demonstrated that large-scale, multicenter translational research using frozen samples was feasible when the sample shipment and Web-based data collection were well organized. The results of the study will identify the predictors of benefit from each 5-FU derivative, and will contribute to establish the “personalized therapy” in adjuvant chemotherapy for colon cancer.

**Trial registration:**

ClinicalTrials.gov: NCT00918827, UMIN Clinical Trials Registry (UMIN-CTR) UMIN000002013

## Background

Colorectal cancer (CRC) is the second most common cancer and the third most fatal cancer in Japan. In particular, colon cancer has been increasing in recent years [1,2]. Postoperative adjuvant chemotherapy for patients with stage III colon cancer is internationally accepted as a standard care to improve survival, and there are several options as treatment regimens. The Guidelines 2010 for the Treatment of Colorectal Cancer published by the Japanese Society for Cancer of the Colon and Rectum (JSCCR) [3] recommend four regimens as adjuvant therapy for stage III disease: 5-fluorouracil (5-FU)/leucovorin (LV), UFT/LV, capecitabine, 5-FU/LV (or capecitabine) + oxaliplatin. In addition, S-1 [4] or S-1 + oxaliplatin [5] are used on an experimental basis.

In Japan, oral 5-FU derivatives (5-FUs) have been preferred because of their convenience, leading to the development of several oral 5-FUs with different properties. UFT (Taiho Pharmaceutical Co., Ltd., Tokyo, Japan) is a combination drug of tegafur, a prodrug of 5-FU, and uracil, an inhibitor of dihydropyrimidine dehydrogenase (DPD) that is a degradative enzyme for 5-FU, in a molar ratio of 4:1 [6]. Concomitant use of folic acid derivative LV with UFT promotes stabilising the ternary complex and augmenting the inhibition of thymidylate synthase (TS) by 5-FU. Capecitabine (Chugai Pharmaceutical Co., Ltd., Tokyo, Japan) is designed to be specifically transformed from 5’-deoxy-5-fluorouridine (5’-DFUR) to 5-FU by thymidine phosphorylase (TP), which is in higher concentrations in tumor tissues than in normal tissues, with the aim of reducing the gastrointestinal and hematological toxicities of 5-FU [7]. S-1 (Taiho Pharmaceutical Co., Ltd.) combines tegafur, gimeracil and oteracil, in a molar ratio of 1:0.4:1. Gimeracil, a DPD inhibitor, is about 200-fold more potent than uracil. Oteracil inhibits the conversion of 5-FU to active metabolites in the gastrointestinal tract, resulting in reduction of gastrointestinal toxicity of 5-FU [8]. These preparations have different profiles of adverse event (AE). UFT is reported to be often associated with liver dysfunction in Japanese patients [4,9]. A most common AE of capecitabine is hand-foot syndrome (HFS) [10]. S-1 often causes mild hematological toxicity and fatigue [4].

Multiple options are available for adjuvant therapy with 5-FUs for colon cancer, however, which drug is the most appropriate for each patient has not been established. The efficacy of 5-FUs is reported to be related to the expression of enzymes that correlate to 5-FU activation/metabolism in tumors and/or blood. Ichikawa et al. [11] reported that of 37 patients with metastatic CRC, UFT/LV was useful in those with low tumor mRNA expression of TS, DPD, and orotate phosphoribosyl transferase (OPRT). Nishimura et al. [12] disclosed that from analysis of 88 patients with Dukes B/C CRC who received 5’-DFUR therapy, those with high tumor TP protein expression in enzyme-linked immunosorbent assay (ELISA) showed better prognosis. On the other hand, TP is also known as a platelet-delivered endothelial cell growth factor, and it has been reported that metastatic CRC with high tumor mRNA levels of TP had poor prognosis [13].

Prediction of efficacy in 5-FUs treatment has been studied [14] but has not been clinically applied yet, because of no large prospective studies or because of difficulty in measurement as point of versatility and cost. “Personalized therapy”, which means to select the best option from different treatments based on individual patients’ factors, could improve not only the efficacy but also the safety, patient’s quality of life and cost-effectiveness in adjuvant therapy for colon cancer.

We therefore conducted a prospective cohort study named the B-CAST study (Biomarker-cohort study of adjuvant chemotherapy for stage III colon cancer), to identify the patients who benefit from adjuvant chemotherapy with each 5-FU derivative, through evaluating the relationships between tumor biomarker expression and treatment outcome.

## Methods/design

### The design of study

This study is a multicenter, prospective cohort study evaluating the relationships between tumor biomarker expression and treatment outcome of adjuvant chemotherapy in patients with stage III colon cancer. In this paper, the disease stage is described by the 7th UICC–TNM classification system.

Six candidate biomarkers are selected for this study: TP, DPD, TS and OPRT as key enzymes correlating to 5-FU activation/metabolism [11-14], and epidermal growth factor receptor (EGFR) and vascular endothelial growth factor (VEGF) as promising markers which are correlated to tumor proliferation and/or metastasis of CRC [15-18] and which are already in clinical use as targets of the molecular-target drug.

The frozen tumor samples from surgical specimen of patients with stage III colon cancer who receives postoperative adjuvant chemotherapy are examined. Protein expression of TP, DPD, EGFR and VEGF are evaluated using ELISA, and mRNA expression of TP, DPD, TS and OPRT are evaluated using reverse transcription polymerase chain reaction (RT-PCR).

**Figure 1:**
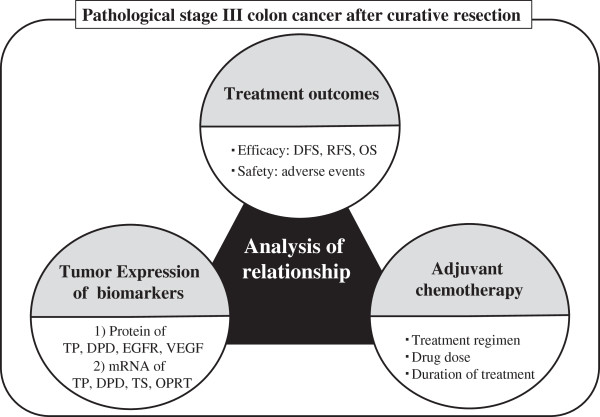
Study concept of the B-CAST study.

The patients’ clinical data including demographic and pathological characteristics [19], regimens, drug doses and treatment duration of adjuvant chemotherapy, and treatment outcomes including AEs and survivals are collected (Tables 1, 2). Then, relationships among the protein/mRNA expression and the patients’ clinical data are analyzed (Figure 1). The primary endpoint is disease free survival (DFS), and secondary endpoints are relapse free survival (RFS), overall survival (OS), and incidence and severity of AEs.

**Table 1:** A list of collected demographic and pathological data at enrollment

**Items**	**Category**
At tentative enrollment:	
- Age at surgery	
- Gender	Male, Female
- Date of surgery	
- Tumor location	C, A, T, D, S, RS
- Scope of LN dissection*	D3, D2, D1, D0
- Surgical approach	Open surgery, Laparoscopic surgery
- Interval between bowel resection and storage at freezer	<1 hour, 1–3 hours, > 3 hours
At final enrollment:	
- Histological type*	pap, tub1, tub2, por1/por2, muc, sig
- Depth of tumor invasion	T1, T2, T3, T4a, T4b
- Lymphatic invasion*	ly0, ly1, ly2, ly3
- Venous invasion*	v0, v1, v2, v3
- No. of LN examined	
- No. of metastatic LN	
- Degree of LN metastasis*	N1, N2, N3

*: Japanese Classification of Colorectal Carcinoma, Second English Edition [ref no. [19].

LN: lymph node.

**Table 2:** A list of collected data regarding adjuvant chemotherapy

**Items**	**Category**
Treatment regimen	
- Drug names	
- Initial dose of each drug (mg/body/day)	
Duration of treatment (days)	
- Date of starting chemotherapy	
- Date of finishing chemotherapy	
Reason for finishing chemotherapy	Completed the scheduled chemotherapy,
	Discontinued by AEs,
	Discontinued by the other reason,
	Withdrew informed consent
Relative dose intensity	≥75%, 75%>−≥50%, >50%
Most severe grade of each AE (with the date of development)	
- Hand foot syndrome	None, G1, G2, G3
- Leukocytopenia	None, G1-2, G3, G4, G5
- Neutropenia	None, G1-2, G3, G4, G5
- Diarrhea	None, G1-2, G3, G4, G5
- Vomiting	None, G1-2, G3, G4, G5
- Anorexia/Nausea	None, G1-2, G3, G4, G5
- Increased AST/ALT	None, G1-2, G3, G4, G5
- Hyperbilirubinemia	None, G1-2, G3, G4, G5
- Other AEs*	G3, G4, G5

*: To be reported only when the severity is grade 3 or higher.

AE: adverse event.

G: grade (the National Cancer Institute Common Terminology Criteria for Adverse Events version 3.0).

AST: aspartate aminotransferase.

ALT: alanine aminotransferase.

The registration period is from April 2009 to March 2012, and the follow-up period is 5 years from the enrollment of the last subject.

### Selection of candidate patients

**Figure 2:**
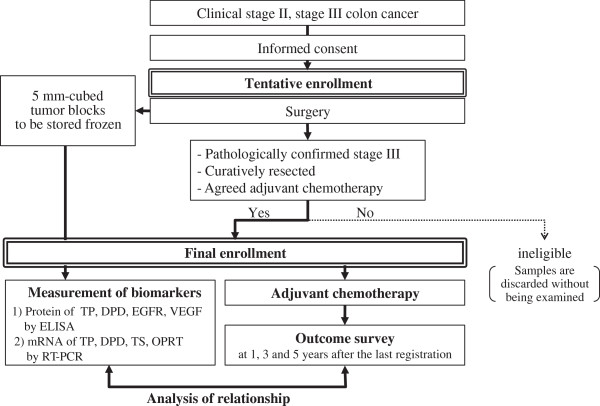
Study schema of the B-CAST study.

Candidate patients are asked to give informed consent prior to surgery (Figure 2). Main inclusion criteria are as follows:

1) Clinical stage II or III colon cancer

2) Pathologically confirmed adenocarcinoma

3) Curatively resectable

4) No prior chemotherapy or radiotherapy for colon cancer

5) Adequate general condition for postoperative adjuvant chemotherapy

6) No other active malignancies (i.e. diagnosed within 5 years)

7) No contraindication to 5-FUs

Patients who have given written informed consent are tentatively enrolled at the Foundation for Biomedical Research and Innovation, Translational Research Informatics Center (FBRI-TRI) using a Web-based enrollment system and a “sample number” is issued. A list of collected information at tentative enrollment is shown in Table 1.

### Collection/storage/submission of tumor samples

Two 5 mm-cubed tissue blocks are taken from the land wall of the primary tumor immediately after surgery, and separately put in 2 predefined tubes. One for protein analysis is put in the blank tube and placed in a freezer. The other for mRNA analysis is immersed in RNAlater® (Life Technologies Japan Ltd., Tokyo, Japan) in the tube. After rapped with a small air-cushion bag, the tube is placed in a freezer such that the tissue block is gradually moistened with RNAlater® before freezing.

In a preliminary experiment using xenografts, there was no difference in the sample stabilities between the samples stored in a deep freezer (−80°C) and those stored in a freezer for general use (usually at −20°C) for up to 8 weeks (data not shown). From these observations, samples are allowed to be stored frozen in a freezer for general use for up to 8 weeks.

Each tube is labeled with the “sample number” issued at the tentative enrollment. The samples are collected by SRL Medisearch Co., Ltd. (SRLM) (Tokyo, Japan), a commercial laboratory company with a nationwide network. The collected samples are temporarily stored in a −80°C depository at SRLM, and then sent to the measurement facility (Kamakura Research Laboratories, Chugai Pharmaceutical Co., Ltd., Kanagawa, Japan).

### Final enrollment

After a pathological examination is completed, patients who meet all of the following criteria are finally enrolled in the study (Figure 2):

1) Pathologically confirmed stage III colon cancer

2) Judged to be curatively resected (R0 resection)

3) Agreed to receive postoperative adjuvant chemotherapy containing 5-FUs

A list of collected information at final enrollment is shown in Table 1. A “final registration number” is issued for each patient after confirming eligibility at FBRI-TRI.

### Measurement of biomarkers

The samples from patients who are eligible for “final enrollment” are examined in the measurement facility in accordance with the Standard Operating Procedures. The samples from ineligible patients (i.e. pathological stage II) are discarded without being examined.

#### Measurement of protein expression

The tumor block is wholly homogenized using an analysis buffer and then centrifuged. Using the resulting supernatant, the protein expressions of TP, DPD, EGFR, and VEGF are evaluated by ELISA. For TP and DPD, the methods established by Chugai Pharmaceutical Co., Ltd. are used [20,21]. For EGFR and VEGF, Human EGFR Duo Set ELISA kit and Human VEGF Quantikine ELISA Kit (R&D Systems Inc., Minneapolis, MN, USA) are used, respectively. All measurements are performed in duplicate, and the mean is used for analyses.

#### Measurement of mRNA expression

The tumor block is wholly homogenized using an analysis buffer. The total RNA is extracted from the homogenate using Sepasol®-RNA I Super G (Nacalai Tesque Inc., Kyoto, Japan) and precipitated with ethanol. The RNA quality is determined using Experion™ Automated Electrophoresis Station (Bio-Rad Laboratories, Inc., Hercules, CA, USA) and assessed based on the RNA Quality Indicator which is calculated from the observed and reference values at 18S and 28S peaks using Experion software (Bio-Rad Laboratories, Inc.). If the RNA quality is adequate, cDNA is synthesized using 5 μg of total RNA.

The mRNA expressions of TP, DPD, TS, and OPRT are evaluated by real-time PCR (LightCycler®480 Real-Time PCR System, Roche Diagnostics, Mannheim, Germany) with commercially available primer/probe sets (Nihon Gene Research Laboratories Inc., Sendai, Japan). Using glyceraldehyde-3-phosphate dehydrogenase (GAPDH) as the internal standard, the mRNA expression of the target gene is quantified. All measurements are performed in duplicate, and the mean will be used for analyses.

After all eligible samples are examined, the measurement results are submitted to FBRI-TRI data center. They are not reported to the attending physician, so that the subsequent treatment is not affected by the measurement results.

### Treatment

#### Adjuvant chemotherapy

The study protocol does not define treatment regimens and schedule of hospital visits during chemotherapy. When the chemotherapy is finished, the following information regarding adjuvant chemotherapy is reported using a Web-based case report system: treatment regimen, dose of each drug, duration of treatment, relative dose intensity, and AEs. Details of collected information are shown in Table 2.

#### Surveillance for relapse

**Figure 3:**
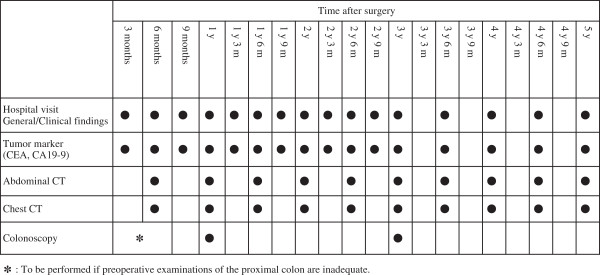
Recommended surveillance schedule in the Japanese guidelines.

Surveillance for relapse in accordance with a schedule described in the JSCCR Guidelines is recommended [3] (Figure 3). Web-based outcome reporting is conducted 3 times, at 1, 3, and 5 years from the last registration (Figure 2). Cases of relapse and/or other malignancy are required to be reported with the date of confirmation and the site. Cases of death are required to be reported with the date and cause of death. For surviving patients, the date of the last confirmation of survival is required to be reported.

### Treatment outcomes

#### Efficacy

DFS (primary endpoint) is defined as the time to relapse, other malignancies or death, whichever comes first. Patients alive and free of relapse or other malignancies are censored at time of the last follow-up. RFS is defined as the time to relapse or death. Patients alive and free of relapse are censored at time of last follow-up. OS is defined as the time to death. The intervals are calculated from the date of surgery.

#### Safety

The types and severities of AEs are evaluated according to the National Cancer Institute Common Terminology Criteria for Adverse Events version 3.0 (National Cancer Institute, Bethesda, MD, USA). HFS is assessed in accordance with the assessment criteria for HFS [22]. The most severe grade of each AE during whole treatment period is reported. The following AEs are required to be reported as “priority survey items”: leukocytopenia, neutropenia, HFS, diarrhea, vomiting, anorexia/nausea, increased aspartate aminotransferase (AST), increased alanine aminotransferase (ALT), and hyperbilirubinemia. Other AEs should be reported when the severity is grade 3 or higher.

### Statistical matters

#### Target sample size calculation

As no studies evaluating the relationship between tumor expression of 5-FU activation/metabolism enzymes and prognosis numerically through the hazard ratio (HR), the necessary sample size was calculated through simulation using previous data on tumor TP.

In 6,731 Japanese colon cancer patients, mean logarithmic tumor TP (log TP) determined by ELISA was 4.31 with a standard deviation (SD) of 0.61, with a normal distribution (unpublished data). We thus assumed that log TP in the present study population follows a normal distribution with a similar mean and SD. The protocol preparation committee concluded that capecitabine, the efficacy of which may be affected by tumor TP [12], is recommended to be chosen when increased tumor TP produces a 7% increase in 5-year DFS rate. In the present study, therefore, the HR of 1-unit decrease in log TP for DFS was estimated to be 1.3 (equivalent to a 7% decrease in 5-year DFS rate).

On the assumption that DFS would follow an exponential distribution with a 5-year DFS rate of 70% [3,23,24] in this study with a registration period of 2 years*, a follow-up period of 5 years, and patient enrollment following a uniform distribution over 2 years* from the start of the study, a proportional hazard model with log TP as the covariate and log HR as the true regression coefficient was applied to produce simulated data with 1,000 iterations for each given HR and sample size. The proportional hazard model was fitted to these simulated data to test the significance of the regression coefficient at a two-sided significance level of α=0.05 based on the null hypothesis that “tumor TP is not correlated with DFS.” Statistical Analysis System version 9.1 (SAS institute Inc., Cary, NC, USA) was used for simulation.

The simulation results showed that a sample size of 950 with a HR of 1.3 would provide a power of 80% or more. With an estimated dropout rate of approximately 5%, the target sample size of 1,000 was estimated to evaluate the relationship between tumor TP and DFS in patients treated with capecitabine. Since three regimens (capecitabine, UFT/LV, and intravenous 5-FU/LV, respectively) were used, the total target sample size of 3,000 was needed to ensure that similar analyses would be performed for each of the three regimens.

*: Because of the delayed cumulative pace of patient enrollment, the registration period was extended in February 2011, from 2 years to 3 years.

#### Analysis plan

In each treatment group, the Cox proportional hazard model with covariate as tumor TP is primarily performed to evaluate significant relationship with DFS. Patients receiving oxaliplatin containing regimens will be evaluated separately from those with 5-FU derivatives alone. At 3 years and 5 years after the enrollment of the last subject, an interim and final analysis is conducted using the Cox proportional hazard model as mentioned above, respectively. The abovementioned analyses for tumor TP, the primary variables, are also performed for other measured biomarkers. Since this is an observational study (not an experimental study), no adjustment for multiplicity is applied.

Secondarily, RFS and OS are also studied in the same manner as DFS. The relationships between expression of biomarkers and AEs in each treatment group are also evaluated by applying a logistic regression model.

And as exprolatory analyses, univariate and multivariate relationships among survivals of each treatment group, expression of biomarkers and patient characteristics will be evaluated using the Cox proportional hazard model.

### Ethical matters

This study is conducted in accordance with the “Declaration of Helsinki” and “Ethical Guidelines for Clinical Research”, and has been approved by the Institutional Review Boards of each participating institute. Written informed consent is obtained from all patients before enrollment.

## Discussion

The B-CAST study is conducted to prospectively evaluate the relationships between tumor biomarker expression and treatment outcome in large sample size of stage III colon cancer patients who received adjuvant chemotherapy, in order to identify the patients who benefit from each 5-FU derivative. A total of 2,128 patients from the 217 institutions were finally enrolled between April 2009 and March 2012. Follow-up will be completed in March 2017.

In this study, frozen tumor samples are used to measure the biomarkers for better quality and quantity of protein/mRNA than those of paraffin-embedded specimens. And in considering of the practical versatility, homogenates of whole specimens of tumor mass, not micro-dissected cancer tissues, are used. The most translational research has conventionally been conducted as mono-institutional research because of difficulty in sample shipment and storage, especially when using frozen samples, resulting in underpowered studies with a small sample size. The B-CAST study demonstrated that large-scale, multicenter translational research using frozen samples was feasible when the sample shipment and Web-based data collection were well organized.

The results of the study will provide the large database of tumor biomarker expression of colon cancer, and will identify the predictors of benefit from each 5-FU derivative. These observations will contribute to establish the “personalized therapy” in adjuvant chemotherapy for colon cancer.

## Abbreviations

CRC: Colorectal Cancer; JSCCR: Japanese Society for Cancer of the Colon and Rectum; 5-FU: 5-fluorouracil; LV: Leucovorin; 5-FUs: 5-FU derivatives; DPD: Dihydropyrimidine Dehydrogenase; TS: Thymidylate Synthase; 5’-DFUR: 5’-Deoxy-5-Fluorouridine; TP: Thymidine Phosphorylase; AE: Adverse Event; HFS: Hand-Foot Syndrome; OPRT: Orotate Phosphoribosyl Transferase; ELISA: Enzyme-Linked Immunosorbent Assay; EGFR: Epidermal Growth Factor Receptor; VEGF: Vascular Endothelial Growth Factor; RT-PCR: Reverse Transcription Polymerase Chain Reaction; DFS: Disease Free Survival; RFS: Relapse Free Survival; OS: Overall Survival; FBRI-TRI: Foundation for Biomedical Research and Innovation, Translational Research Informatics Center; SRLM: SRL Medisearch Co., Ltd.; GAPDH: Glyceraldehyde-3-Phosphate Dehydrogenase; AST: Aspartate Aminotransferase; ALT: Alanine Aminotransferase; HR: Hazard Ratio; Log TP: Logarithmic tumor TP; SD: Standard Deviation

## Competing interest

B-CAST study [TRICC0807] is conducted as a part of joint research of Tokyo Medical and Dental University and the Foundation for Biomedical Research and Innovation (FBRI) on construction of foundation for large-scale translational research, with funding from FBRI. FRBI is financed by manufacturers and distributors of the drugs, but there are no competing interest between these companies and the investigators that require disclosure in connection with the study.

MI has received consulting fees from Taiho Pharmaceutical Co. Ltd., Bristol-Myers Squibb and Merck Serono Co. Ltd; honoraria from Taiho, Chugai Pharmaceutical Co. Ltd., and Yakult Honsha Co. Ltd.

K.Kotake has received consulting fees from Taiho and Chugai; honoraria from Taiho, Chugai, Bristol-Myers, Merck Serono, Yakult Honsha, Otsuka, Daiichi Sankyo Co. Ltd., and MSD K. K.

GN has received honoraria from Chugai.

NT and WI have received honoraria from Taiho and Chugai.

KT has received honoraria from Takeda Pharmaceutical Co. Ltd.

TW has received honoraria and research funding from Taiho, Chugai, Daiichi Sankyo, Yakult Honsha, Bristol-Myers, Merck Serono, and Takeda.

MM has received honoraria from Taiho.

YK has received honoraria from Chugai and Sanofi-Aventis K.K.; research funding from Chugai.

IH has received honoraria and research funding from Taiho, Chugai, and Daiichi Sankyo.

TF, and K. Kondo, AK, MK, MO, and KM have no competing interest.

KS has received consultant fees, research funding and honoraria from Taiho, Chugai, Takeda, Yakult Honsha, Daiichi Sankyo, Bristol-Myers, Merck Serono, and Pfizer Co. Ltd.

## Authors’ contributions

MI, as a task manager, participated in entire coordinating of the study, design and writing of the protocol, data collection, data analysis, data interpretation, and writing of the manuscript. K. Kotake, GN, NT, WI and KS, as protocol preparation committee, participated in all phases of this study, including design and writing of the protocol, data collection, data analysis, data interpretation, and preparation of the manuscript. KT, TW, TF, K. Kondo, MM, YK, AK, MK, MO, and IH, as steering committee, participated in all phases of this study, including evaluation of the protocol, data collection, data analysis, data interpretation, and preparation of the manuscript. KM, as a chief of statistical analysis, participated in statistical setting of study design and data analysis. All authors reviewed and approved the final manuscript.

## Pre-publication history

The pre-publication history for this paper can be accessed here:

http://www.biomedcentral.com/1471-2407/13/149/prepub
